# Comparison of Del Nido (a different application) and crystalloid blood cardioplegia on arrhythmia and early results

**DOI:** 10.1186/s13019-024-02675-1

**Published:** 2024-04-16

**Authors:** Ferhat Borulu, Ümit Arslan, Eyüp Serhat Çalik, Kaptanıderya Tayfur, Bilgehan Erkut

**Affiliations:** 1 Faculty of Medicine, Department of Cardiovascular Surgery, Ordu University, Ordu, Turkey; 2 Faculty of Medicine, Department of Cardiovascular Surgery, Atatürk University, Erzurum, Turkey

**Keywords:** Del nido cardioplegia, Arrhythmia, Coronary artery bypass surgery

## Abstract

**Background:**

The results of the use of del-Nido(DN) solution using a different method or crystalloid blood cardioplegia in coronary bypass patients were compared. We aimed to investigate the effects on intraoperative and postoperative arrhythmias, arrhythmia durations and early results.

**Methods:**

The study included 175 patients using crystalloid blood cardioplegia (Group 1) and 150 patients using DN solution(Group 2). In the DN group, 75% of the calculated plegia dose was given first. the remaining part was applied by giving from grafts. Intraoperative/postoperative data were compared.

**Results:**

There was no significant difference between the groups in terms of demographic characteristics. Preop troponin level was similar.(*p* = 0.190) However, there was a statistical difference between the postoperative 6th hour.(*p* = 0.001) There was no difference in troponin values at the postoperative 24th hour. (*p* = 0.631) Spontaneous rhythm occurred at the cardiopulmonary by pass (CPB) weaning stage in most of the patients in Group 2 (95.3%). Although the need for temporary pacing was less in Group 2, it was not significant.(*p* = 0.282) No patient required permanent pacing. CPB duration, cross clamp times and intraoperative glucose levels, intensive care follow-up times and hospitalization times were found to be shorter in Group 2. Although the postoperative atrial fibrillation frequency was similar (*p* = 0.261), the time to return to sinus was lower in Group 2.(*p* = 0.001).

**Conclusion:**

The use of DN cardioplegia solution provides significant positive contributions to avoid arrhythmias compared to crystalloid blood cardioplegia. DN solution applied with this method may contribute to reducing the anxieties associated with its use in isolated coronary artery bypass surgery.

## Introduction

One of the most important issues in cardiac surgery is to provide appropriate myocardial protection. Attempts have been made to achieve reversible diastolic arrest by direct administration of a cardioplegic solution to the coronary vessels. Different suggestions and discussions continue regarding this solution [1]. Studies have been conducted to investigate the approiate content, temperature (cold or hot), mode of administration (antegrade or retrograde), and duration. Del Nido (DN) cardioplegia is considered as cardioplegia solutions protecting myocytes. Although it was first used in pediatric cardiac surgery, studies indicating that it can be used in adult cardiac surgery have increased in the following years [2, 3]. The contents of the DN cardioplegic solution are given in Table 1 (with blood cardioplegia). The main difference of this solution from classical cardioplegia is that it contains lidocaine. Lidocaine slows down electrical activity across the cell membrane of myocytes. Thus, it reduces intracellular calcium accumulation, which is the precursor of cell damage, and reduces energy consumption [4]. The mannitol in its content saves the myocardium by acting as an effective cleansing agent for reactive oxygen species (ROS) by making osmotic diuresis. The solution reduces the intracellular accumulation of harmful calcium ions, decreases the energy consumption rate, enables free radical scavanging, and reduces myocardial edema [5, 6]. Its administration as a single dose provides an important advantage in terms of ease of use. A prospective randomized study identified better spontaneous rhythm after operation, less need for defibrillation and inotropic support, and rapid improvement in troponin values as the most important advantages of using the DN cardioplegic solution [7].

**Table 1:** Contents of cardioplegia solutions

Contents	del-Nido Plegia 1000 cc	Blood Cardioplegia 1000 cc
Potassium Chloride (%7,5 1 mEq/L)	26 cc	16 cc
Magnesium Sulphate (%15)	14 cc	-
Sodium Bicarbonate (%8,4 1mEq/l)	13 cc	10 cc
Lidocaine (%2)	6,5 cc	-
Mannitol (%20)	17 cc	-
Blood (cc)	200 cc	800 cc

We conducted this study with the confidence of previous studies. We aimed to examine the effect of using DN cardioplegia compared to classical blood cardioplegia and incidence of arrhythmias and the duration of arrhythmia.

## Materials and methods

### Study design

Two groups were formed in our study, one including 175 patients who were given blood cardioplegia between January 2018 and January 2020 and the other including 150 patients who received DN cardioplegia since January 2020. Patients who underwent cardiopulmonary bypass surgery(CPB) during surgery were included in the study. Demographic characteristics such as age, gender, hypertension, diabetes mellitus, smoking, hyperlipidemia and COPD were compared with EuroSCORE II data (Table 2). CPB duration, cross-clamp time, number of coronary vessels grafted, body temperature, and amount of cardioplegic solution were examined (Table 3). Intraoperative arterial blood gas (ABG) data were analyzed (Table 4). Preoperative and postoperative troponin values and other laboratuary data(Table 5), of the patients in both the groups were evaluated (Table 6). The basis of the study were the presence of arrhythmias, especially atrial fibrillation, which developed during the intraoperative period (arrhythmias occurring and interventions administered until leaving the pump and arriving at the ICU), and their durations in the postoperative period. Arrhythmia follow-up was conducted until discharge. All patient’s’ routine follow-up data were compared.

**Table 2:** Demographic data

Parameters	Group 1 (n: 175)	Group 2 (n: 150)	P value
Age (year)	60,57±9,68	61,47±8,97	0,526*
Gender			
Male (n/%)	141/80,6	109/72,7	0,092**
Female (n/%)	34/19,4	41/27,3	0,112**
BMI ( kg/m^2^)	26,89±3,2	27,94±2,9	0,875*
BSA ( m^2^)	1,95±0,16	1,96±0,18	0,832*
Hypertension (n/%)	56 (%32)	52 (%34,7)	0,611**
DM (n/%)	54 (%30,9)	49 (%32,7)	0,727**
Cigarette (n/%)	50 (%28,6)	41 (%27,3)	0,804**
Hyperlipidemia (n/%)	41 (%23,4)	40 (%26,7)	0,501**
Preop EF (%)	49,94±5,67	50,49±6,51	0,261*
COPD (n/%)	15 (%8,6)	13 (%8,7)	0,976**
PAD (n/%)	4 (%2,3)	5 (%3,3)	0,405**
CRF (n/%)	5 (%2,9)	4 (%2,7)	0,595**
CVD (n/%)	2 (%1,1)	3 (%2)	0,428**
Euroscore II	1,09±0,42	1,3±0,46	0,001*
Emergency Surgery (n/%)	3 (%1,71)	4 (%2,7)	0,661**

BMI: Body Mass Index DM: Diabetes Mellitus PAD: Peripheral Artery Disease CVH: Cerebro Vascular Disease BSA: Body Surface Area COPD: Chronic Obstructive Pulmonary Disease CRF: Chronic Renal Failure EF: Ejection Fraction

* Mann Whitney U Test ** Chi-Square Test

**Table 3:** Intraoperative data

Parameters	Group 1 (n: 175)	Group 2 (n: 150)	P value
CPB time ( min.)	100,01±25,64	89,21±18,97	0,001*
Cross clamp time ( min.)	50,09±15,97	45,02±13,56	0,001*
Number of vessels (n)	3,16±0,81	3,05±0,88	*0,287*
Body temperature (^o^ C)	31,64±0,9	31,65±0,9	*0,841*
Pleji solution amount (ml)	1612,57±229,83	1568,93±177,51	*0,106*
Intraoperative use of inotropes (n)	104 (%59,4)	84 (%56)	*0,282*

*Mann Whitney U Test CPB: Cardio Pulmonary Bypass

**Table 4:** Intraoperative laboratory data

	Initiation of CPB			X-clemp 15.minute			Termination of CPB		
	Group 1	Group 2	*p value*	Group 1	Group 2	*p value*	Group 1	Group 2	*p value*
pH	7,33±0,07	7,32±0,06	*0,223*	7,35±0,06	7,35±0,06	*0,830*	7,35±0,06	7,36±0,06	*0,245*
pO2	244,38±49,9	239,65±58,52	*0,121*	184,5±43,08	187,51±46,58	*0,469*	188,69±28,61	189,17±36,1	*0,984*
pCO2	42,53±5,29	42,78±6,1	*0,638*	38,54±5,81	38,8±6,64	*0,938*	37,26± 5,13	38,37±5	*0,894*
HCO3	21,82±1,59	21,8±2,34	*0,709*	21,37±1,68	20,64±1,96	0,001*	21,28±1,52	20,99±1,58	*0,112*
BE	3,27±1,58	3,45±2,09	*0,393*	3,47±1,55	4,16±1,89	0,001*	3,55±1,47	3,72±1,52	*0,210*
Saturation	99,14±0,55	99,04±0,61	*0,055*	98,82±0,81	98,84±1,13	*0,328*	98,63±6,83	99,03±0,72	*0,356*
NaCl	137,43±2,36	137,47±2,9	*0,919*	138,26±2,37	138,43±2,64	*0,628*	138,1±1,52	139,39±1,83	*0,052*
KCl	4,63±0,55	4,52±0,69	*0,208*	4,92±0,54	4,64±0,68	0,001*	4,91±3,61	4,55±0,48	*0,124*
Glucose	160,83±35,44	155,48±41,08	*0,140*	212,8±40,92	196,38±45,22	0,001*	203,08±38,73	186,28±43,26	0,001*
Calcium	1,34±0,09	1,33±0,14	*0,326*	1,28±0,05	1,27±0,08	*0,122*	1,3±0,08	1,13±1,01	*0,284*
Lactate	1,96±0,82	1,85±0,94	*0,059*	2,68±0,95	2,69±1,7	*0,141*	2,72±0,95	2,76±1,22	*0,560*
Hemoglobin	7,2±1,18	7,16±1,51	*0,848*	7,81±0,86	7,76±1,09	*0,340*	7,89±0,82	7,93±1	*0,926*
Hematocrit	22,68±3,66	22,4±4,11	*0,393*	23,69±2,59	23,94±3,64	*0,437*	23,97±2,6	24,58±2,98	

*Mann Whitney U Test

**Table 5:** Preoperative and postoperative laboratory data

Variables	Group 1 (n: 175)	Group 2 (n: 150)	P value
*Preop Troponin*	78,37±98,17	76,42±106,57	0,190
*Preop BUN*	20,79±6,73	21,42±7,05	0,214
*Preop Creatinine*	0,91±0,21	0,9±0,23	0,232
*Preop HGB*	14,49±1,63	14,53±1,92	0,787
*Preop WBC*	8,39±1,18	8,37±1,28	0,838
*Preop PLT*	258,66±63,34	262,78±70,14	0,731
*Preop CK*	82,95±57,51	87,81±56,55	0,146
*Preop CKMB*	34,56±21,53	34,77±21,8	0,658
*Preop LDH*	258,22±35,36	263,31±31,7	0,282
*Preop INR*	1,07±0,06	1,06±0,05	0,193
*Postop 6 h Troponin*	838,66±371,89	731,04±374	0,001*
*Postop 24 h Troponin*	441,65±262,54	444,35±329,32	0,631
*Postop Day 1 BUN*	25,2±6,35	24,58±7,02	0,251
*Postop Day 1 Creatinine*	1,15±1,63	1,01±0,25	0,100
*Postop Day 1 HGB*	8,68±0,73	8,7±0,85	0,982
*Postop Day 1 WBC*	14,65±2,52	13,96±2,69	0,485
*Postop Day 1 PLT*	171,17±34,97	176,28±36,3	0,343
*Postop Day 1 KCL*	4,22±0,28	4,29±0,4	0,201
*Postop Day 1 CA*	8,61±0,54	8,59±0,5	0,820

BUN: Blood Urine Nitrojen HGB: Hemoglobin WBC: White Blood Cell CVH: Cerebro Vascular Disease PLT: Platelet CA: Calcium CK: Creatin Kinase LDH: Lactate Dehydrogenase INR: International Norm Ratio

* Mann Whitney U Test

**Table 6:** Postoperative follow-up data

Parameters	Group 1 (n: 175)	Group 2 (n: 150)	p value
Duration of mechanical ventilation (hour)	7,31±4,29	6,92±5,84	0,001*
ICU stay time (hours)	63,27±21	52,33±14,76	0,001*
Hospitalization time (days)	7,17±1,51	6,7±1,15	0,001*
Postoperative EF (%)	50,4±5,01	50,78±6,04	0,441
Postop mechanical support ( n/%) IABP	9 (%5,1)	8 (%5,3)	0,819
ECMO	2 (%1,3)	3 (%1,7)	0,572
Postop inotrope use ( n/%)	36 (%20,6)	27 (%18)	0,559
Postop major complication ( n/%)	14 (%8)	10 (%6,7)	0,819

Mann Whitney U Testi *

ICU: Intensive Care Unit EF: Ejection Fraction IABP: Intra Aortic Balloon Pump ECMO: Extra Corporal Membrane Oxygenator *ES: Erythrocyte Suspension FFP: Fresh Frozen Plasma*

Approval for the conduct of the study was obtained from the ethics committee of our university (Atatürk University Faculty of Medicine Clinical Research Ethics Committee, May 25, 2020; meeting/decision No. 6/60). As the data analysis in the study was conducted retrospectively, informed consent was not obtained from the patients. The study was conducted in accordance with the principles stipulated in the ‘‘Declaration of Helsinki’’.

### Operative details and cardioplegic solutions

A median sternotomy using a left internal mammary artery graft was performed in all patients. According to the number of other grafts, the saphenous vein and radial artery were used. Trillium Affinity NT (Medtronic, Minneapolis, USA) was used as oxygenator; and Terumo Advanced Perfusion System 1 (Michigan, USA) as the perfusion system. Standard cannulation was performed from the atrium and aorta in all patients, and cardioplegia was administered via the aortic root. Pulsatile pump flow was applied in all patients before the cross-clamp was removed. Mild hypothermia was maintained on CPB (32–34 °C) and the body temperature was brought to euthermia after the removal of aortic cross clamp as the CPB was weaned off. In both patient groups, norepinephrine was started first when positive inotropic support was required. Generally, the amount was initially started as 4–5 ml/minute (10–14 µg per minute) or 0.15–0.20 (microgram/kg/minute) and the amount was adjusted according to the situation in blood pressure. When a second inotropic agent was required, adrenaline or dobutamine was used.

In group 1, while the cardioplegic solution was administered, an antegrade dose calculated according to body weight (20 cc/kg) was applied the first time. One-fourth of the first dose was then administered every 20 min antegradely. When the cross-clamp was removed, antegrade washing was performed with warm blood.

In group 2, 75% of the cardioplegic solution calculated according to body weight was given at the first stage. The remaining amount was divided by the number of grafts i.e. after each distal anastomosis, cardioplegia was administered via the grafts at half the rate of the initial application. This protocol was continued after each anastomosis. After the aortic cross-clamp was removed, the cardioplegia cannula was connected to the aortic cannula, and the cardioplegic solution was removed from the myocardium by continuous washing with hot blood. Thus, the removal of cardioplegia solution from the myocardium was facilitated thanks to the hot blood given during the proximal anastomoses. The properties of the solutions are presented in Table 1. After the cross-clamp was removed, proximal anastomoses were performed with a side clamp.

### Postoperative follow-up process

The patients in both groups were shifted to the ICU for elective post-operative ventilation. They were then extubated during routine procedures. A troponin-I test was performed at the 6th and 24th postoperative hours, together with routine laboratory follow-ups. During the intensive care follow-up of all patients, hemogram and electrolytes were monitored 4 times a day. As the main point of the study was to investigate the frequency and duration of arrhythmias, all patients with arrhythmias that affected hemodynamics and received interventions, the durations of the arrhythmias were recorded (Table 7). All necessary electrolyte replacements were performed in all patients with atrial fibrillation in the postoperative period, in accordance with their laboratory data. After being admitted to the ward, the patients were monitored for vital signs and rhythm with a monitor, as in intensive care, for another 24 h. When palpitations occurred in the following period, electrocardiography was performed and rhythm disorders were detected. First, a beta-blocker (5-mg metoprolol) was administered to the patients diagnosed with atrial fibrillation, followed by amiodarone infusion. The drains in the patients were removed when the drainage volume was less than 100 cc/day.

**Table 7:** Intraoperative and postoperative arrhythmias

Paremeters	Group 1 (n: 175)	Group 2 (n: 150)	p value
Intraoperative VF (n)	149 (%85,1)	4 (%2,7)	0,001*
Intraoperative pacemaker need (n)	10 (%5,7)	4 (%2,7)	0,282
Intraoperative spontaneous rhythm (n)	16 (%9,1)	143 (%95,3)	0,001*
Postoperative AF (n)	30 (%17,1)	19 (%12,7)	0,261
Time to return of postoperative AF to sinus rhythm (hours)	10,87 ± 3,29	7,84 ± 2,34	0,001**
Postoperative VF, VT	2 (%1,1)	2 (%1,3)	0,629

Chi-Square Test * Mann Whitney U Test * * AF: Atrial Fibrillation VF: Ventricular Fibrillation VT: Ventricular Tachycardia

### Statistical analyses

The NCSS (Number Cruncher Statistical System) 2007 (Kaysville, Utah, USA) program was used for the statistical analysis. While evaluating the study data, descriptive statistical methods (mean, standard deviation, median, frequency, ratio, minimum, and maximum) and the distribution of the data were evaluated using the Shapiro-Wilk test. The Mann-Whitney *U* test was used to compare two groups whose quantitative data did not show a normal distribution. A chi-square analysis was used to determine the relationship between the qualitative data. Significance was evaluated at *p* < 0.05.

## Results

Total 325 patients were included in the study. The patients’ demographic characteristics, body mass index values, and preoperative ejection fractions were similar between the two groups (Table 2).

The patients’ operational and postoperative follow-up data are presented in Tables 3 and 4. Cross-clamp times were significantly shorter in the DN group (*p* = 0.001, group 1: 50.09 ± 15.97 min vs. group 2: 45.02 ± 13.56 min). The CPB times (89.21 ± 18.97 min) were also statistically significantly lower in the DN group (*p* = 0.001). The lowest intraoperative body temperatures were similar between the groups (group 1: 31.64^o^C ± 0.9 ^o^C vs. group 2: 31.65 ^o^C ± 0.9 ^o^C). Although the amount of cardioplegic solution used was less in the DN group, the difference was not statistically significant (group 1: 1612.57 ± 229.83 cc vs. group 2: 1568.93 ± 177.51 cc, *p* = 0.106). A significantly higher rate of spontaneous rhythm was observed in patients in the DN group after removal of the cross clamp (*p* = 0.001). The patients in the group given the conventional cardioplegia required more defibrillation (*p* = 0.001). The need for temporary pacemaker support was lower in the DN group, although not statistically significantly (*p* = 0.282; Table 6). Although the number of patients who used inotropes intraoperatively (time from discontinuing CPB to sternal closure) was lower in the DN group, the difference was not statistically significant (*p* = 0.282). No significant differences in blood gas parameters, except for blood glucose level, were observed at the pump inlet, the 10th minute of pumping, and the discontinuing CPB. The blood glucose level was lower in the DN group (*p* = 0.001).

Considering the postoperative data, the durations of mechanical ventilation were similar between the groups, although the length of ICU stay was shorter in the DN group, the difference was not statistically significant. The intensive care follow-up times were lower in the DN group. No significant differences were found in the amount of postoperative drainage, amount of blood transfusion, number of patients who used inotropic support, and postoperative laboratory data. Only the 6th-hour postoperative troponin-I values were lower in the DN group (*p* = 0.001). However, the troponin-I value at the 24th hour was similar between the groups (*p* = 0.631). We found no significant difference in the number of mechanical support devices (intra-aortic balloon pump and extracoronal circulation support) needed for the patients in the postoperative period. Examining the arrhythmias that formed the basis of this study, the number of patients with atrial fibrillation in the postoperative period was lower, but this was not statistically significant. A significant reduction in the time to return to sinus rhythm was observed in patients with atrial fibrillation (*p* = 0.001). No significant difference was found in terms of other rhythm disorders (supraventricular tachycardia and ventricular fibrillation) that may affect hemodynamics and require intervention in the postoperative period (*p* = 0.629). No significant differences were found in the incidence of major complications (e.g., renal failure, cerebrovascular accident, severe bleeding, and revision) and death during the postoperative follow-up period (*p* = 0.819).

## Discussion

Providing myocardial protection of the heart during cardiac operation is one of the most important issues. However, no consensus has been reached as to the form and content of the cardioplegic solutions used for myocardial protection [6]. Reducing the cross-clamp and CPB times combined with myocardial protection is another important issue [8]. There are efforts to reduce these times using various methods. In our literature review, we found significantly shorter cross-clamp and CPB times [5].

Serious concerns have emerged about the use of DN cardioplegia in coronary artery disease, especially in multivessel patients [9]. Although retrograde cardioplegia provides an effective solution in patients receiving conventional blood cardioplegia, this causes confusion, as DN cardioplegia is a one-time and antegrade application. Another important contribution of avoiding the need for retrograde cardioplegia is the protection from complications (e.g., coronary sinus injury and bleeding from the entry site in the atrium) that may be encountered during retrograde cardioplegia cannula placement. As supported by our study, appropriate cardiac arrest can be achieved with adequate myocardial protection without the need for retrograde cardioplegia. In our clinic, in DN cardioplegic application for multivessel patients, after the first antegrade dose, a solution of one quarter of the first dose is given via the anastomosed grafts. Thus, the distribution of the cardioplegic solution to the whole heart is facilitated.

Studies have shown that the DN cardioplegic solution provides safe myocardial protection for patients undergoing coronary revascularization [7, 10].

DN cardioplegia is a solution primarily developed to protect the immature myocardium, which is vulnerable to reperfusion injury. However, later studies have shown that it also provides functional improvements in elderly hearts [11, 12]. With our confidence in these studies, we have used the DN cardioplegic solution in our clinic for the last 2 years. Although we found no significant differences in the postoperative period data in terms of ejection fraction in our study, the low troponin-I values in the postoperative follow-ups were consistent with most reports in the literature. Ziazadeh et al., in their study, suggested that contrary to this view, troponin values were not low in groups given DN cardioplegia [13]. In our study, in terms of intraoperative arrhythmias, which is the main subject of our study, the need for ventricular fibrillation and temporary placement of a pacemaker after cross-clamp removal was significantly lower in the DN group. The spontaneous rhythm formation rate was significantly high. Since there was no significant difference in the intraoperative body temperature, blood gas data and electrolyte data of the patients, our opinion was strengthened that this difference was due to the effect of the cardioplegia solution. Many studies have reported a serious decrease in ventricular fibrillation rate [8, 14, 15]. The possibility of subepicardial necrosis occurring in the epicardium is also reduced, as there is less need for defibrillation. This will contribute to the reduction of myocardial damage [16]. The lower cardiac enzyme levels in the postoperative period and incidence of intraoperative ventricular arrhythmias suggest that the DN cardioplegic solution is effective for myocardial protection. The components of the DN cardioplegic solution effectively provides myocardial protection. Mannitol is effective in scavenging free radicals and reducing edema owing to its hyperosmotic property [17]. With the effect of lidocaine in the solution, the entry of calcium and sodium into the cell decreases. Lidocaine acts as a depolarizing agent by inhibiting sodium channels [18]. Another important component of the DN cardioplegic solution is magnesium, which is used as an electrolyte that has been proven to increase myocardial recovery by blocking calcium channels [19, 20].

It is thought that administering cardioplegia through blood improves oxygen distribution and creates ideal oncotic pressure. In addition, the presence of blood contributes to good maintenance of pH. We think that the lack of difference between intraoperative hemoglobin and hematocrit values in our study contributes positively to minimizing the effects of hemodilution.

When arrhythmias were examined in our study, DN cardioplegic solution showed positive effects in terms of both intraoperative spontaneous rhythm formation and postoperative arrhythmias (frequency and duration of atrial fibrillation, ventricular tachycardias) [21, 22]. It can do this with special ingredients such as mannitol, lidocaine and magnesium. In our study, we did not analyze each etiological factor in rhythm disorders. However, since other follow-up parameters are similar, it suggests that plegia solutions are effective in arrhythmia differences. With studies to be conducted with larger patient groups, the results on this subject may become stronger. In our study, the frequency of atrial fibrillation in the postoperative period was consistent with that reported in the literature [23]. Although the frequency of atrial fibrillation in the postoperative period was lower in the DN group, the difference was not statistically significant. However, the time to return to sinus rhythm was found to be significantly shorter in the patients with atrial fibrillation. Return to sinus rhythm in a shorter time is considered a positive feature of DN cardioplegia. Although studies have reported that atrial fibrillation is less common, this difference was not statistically significant in our study. Although the majority of studies suggest that atrial fibrillation is less common in patients using DN, Timek et al. found that atrial fibrillation is more common in their study [24]. We think that the many etiological factors of atrial fibrillation are the reason for these differences. In addition, it is a positive situation that none of the patients who underwent DN cardioplegia did not experience a significant ventricular arrhythmia in the early postoperative period.

As in many studies in the literature, no significant difference was found between the groups in terms of durations of mechanical ventilation, major complications and discharge times in our study [23, 25]. We think that one reason for the longer intensive care follow-up times in patients treated with conventional cardioplegia is the longer time required for the follow-up of developing arrhythmias and for the recovery of these arrhythmias. Extended ICU stay of the patient until after the arrhythmia has improved is effective in this regard. The absence of significant differences between the groups in terms of the amount of drainage, the amount of blood transfusion, the number of patients using inotropic support, and the postoperative laboratory data is similar to and supportive of the studies on the safe use of this cardioplegic solution, at least in isolated coronary bypass procedures.

Although a study conducted in the Cleveland Clinic in 2014 [2] did not recommend the use of DN cardioplegia for isolated coronary bypass applications, reassuring findings were presented in different studies conducted later [15, 23, 26]. Owing to the lack of prospective studies with large numbers of patients, the concerns of some clinics about this issue could not be eliminated. However, as shown in many studies, DN cardioplegia has become an increasingly preferred choice in clinics, as it allows for the shortening of the clamping and operation times and the completion of cardioplegia at one time. In a large-scale study, Guajardo Salinas et al. showed that application of DN cardioplegia provides an important economic advantage and other clinical benefits [16]. In this study, our findings showed that the DN cardioplegic method can be used safely in patients undergoing isolated coronary bypass.

### Limitations

Between the groups, the DN group had shorter cross-clamp and CPB times. In this study, whether the shortness of these times or the DN content was effective in reducing the frequency and duration of postoperative atrial fibrillation was not analyzed. The basis of the study was the formation of spontaneous sinus rhythm at the termination of the CPB without defibrillation. However, the similarity of other laboratory data in the postoperative period contributed to our interpretation of this issue.

The follow-up data of the patients until discharge were compared, but owing to the difficulty of accessing their retrospective data, medium- and long-term data could not be compared. Other parameters were not compared in terms of ICU length of stay and the reasons for the shorter hospital stay in the DN group. All parameters that might have caused the differences were not compared. AF and the related additional treatments are thought to be effective in this process.

The etiology of arrhythmias experienced by patients in the intraoperative and postoperative periods is known to be multifactorial. Although the basic variables were similar between the groups, conditions such as cardioplegia pressure during infusion, postoperative ventilation strategies, antibiotics and infection, etc. were not compared in our study.

Since the study is a retrospective study, a comparison with the application of blood cardioplegia from grafts could not be made.

As only DN cardioplegia has been used in our clinic after January 2020, the patients in the two groups did not undergo operation in the same period.

## Conclusion

DN cardioplegia applied as a single dose can be used with very good results for rhythm disorders occurring in the intraoperative (spontaneous rhythm formation) and postoperative periods (atrial fibrillation frequency and duration, and ventricular tachycardia) compared with conventional cold blood cardioplegia in patients undergoing isolated coronary revascularization. The fact that these rhythm disorders are less common contributes significantly to the surgical team in the intraoperative and postoperative periods. Larger-scale fully prospective studies will provide a clearer understanding of the contribution of myocardial protection and reduced arrhythmias to the improvement of clinical follow-ups.

## Data Availability

Not applicable.
